# Religious Affiliations and Clinical Outcomes in Korean Patients With Acute Myocardial Infarction

**DOI:** 10.3389/fcvm.2022.835969

**Published:** 2022-03-23

**Authors:** Seok Oh, Ju Han Kim, Kyung Hoon Cho, Min Chul Kim, Doo Sun Sim, Young Joon Hong, Youngkeun Ahn, Myung Ho Jeong

**Affiliations:** ^1^Department of Cardiology, Chonnam National University Hospital, Gwangju, South Korea; ^2^Department of Cardiology, Chonnam National University Medical School, Gwangju, South Korea

**Keywords:** religion and medicine, myocardial infarction, treatment outcome, Republic of Korea, coronary artery disease

## Abstract

**Objective:**

Although religion is expected to have a direct or indirect effect on various aspects of human life, information on the association between religion and acute myocardial infarction (AMI) is inadequate. Hence, in this study, we aimed to investigate the clinical effect of religion on clinical outcomes in patients with AMI.

**Methods:**

A total of 2,348 patients with AMI who were treated by percutaneous coronary intervention (PCI) were enrolled in the study, and they were categorized into two groups depending on their religious belief: religious and non-religious groups. The characteristics and clinical outcomes of both groups were compared.

**Results:**

Compared with the religious group, the non-religious group was younger, included mostly men, was more likely to smoke, and was more likely to be diagnosed with ST-segment elevation myocardial infarction. However, the non-religious group was less likely to have a history of hypertension and tended to receive PCI more quickly with shorter door-to-balloon time. Regarding 1-year clinical outcomes, no differences were found between the two groups.

**Conclusion:**

Despite a growing body of evidence that religious activities have positive effects on human physical health, our results showed a lack of significant differences in 1-year clinical outcomes in patients with AMI irrespective of their religious beliefs.

## Key Message

- Among patients with acute myocardial infarction (AMI) undergoing percutaneous coronary intervention (PCI), the patients in the non-religious group tended to be male and younger and had higher proportions of smokers and a diagnosis of ST-segment elevation myocardial infarction, but a lower proportion of hypertension with shortened door-to-balloon time, when compared with the religious group.
- Although not fully explained, religiosity seems to have short-term favorable effects in patients with AMI undergoing PCI, showing a lower incidence of cardiac death within the first 90 days.
- Regardless of religious beliefs, we found no significant difference in the 1-year clinical outcomes of patients with AMI undergoing PCI.

## Introduction

Cardiovascular disorder is one of the leading causes of death worldwide. Among cardiovascular disorders, acute myocardial infarction (AMI), manifested by myocardial necrosis due to oxygen deficiency, is a major cardiovascular condition that constitutes a high proportion of socioeconomic problems (1, 2). If left untreated, this acute illness could become a real disaster, resulting in life-threatening conditions such as cardiogenic shock, cardiac arrest, and even death. In addition, a considerable number of AMI survivors develop progressive deterioration of the left ventricular ejection fraction (LVEF) with intermittent clinical symptoms or signs of heart failure, also known as ischemic cardiomyopathy. Moreover, AMI may cause changes in the quality of life of patients, thereby significantly reducing the physical, social, and psychological functionalities (3). Therefore, clinical application of multifaceted therapies including optimal pharmacological and reperfusion strategies in patients with AMI is unquestionably important.

Humans come from various cultural backgrounds. Among them, religion can be regarded as the most pervasive and influential factor in cultural dimensions. As one of the social norms, religion is related to various aspects of human behavior, including health. Physical health is indeed liked to several religious doctrines. In Buddhism, according to the teaching of Ahimsa (do no harm), it is recommended to abstain from the meat consumption, which makes believers eat products low in saturated fat or animal fat (4). In the Bible, the apostle Paul said that our bodies are the temple of the Holy Spirit, which may mean that it is crucial to maintain or take care of one’s physical health (5). Confucianism regards health behavior as a beneficial act to sustain dignity, social harmony, and obedience (6). With these teachings, the relationship between religious activities and physical health has been the subject of growing interest (7), and there is a common belief that religious persons will maintain better physical and mental health than non-religious persons through self-management and behavioral restriction associated with enhanced religiousness/spirituality (8). Although such beliefs are still not well explained, the positive effects of religion on people’s health are presumed to be due to several mechanisms, including lifestyle modification, reinforced social support by congregational members, and more positive emotions (9).

South Korea (hereafter referred to as Korea) is one of the most religiously pluralistic nations in the world, despite a high level of ethnic homogeneity (10). Both Buddhism and Christianity (Catholicism and Protestantism) are the major religions of Korea, according to Statistics Korea (11). Buddhism entered the Korean Peninsula at about the fourth century and remained as a significant religion for centuries. Since the establishment of a Catholic community by Peter Yi Seung-hun, a scholar who was baptized in China in the spring of 1,784, which marks the start of the Catholic Church in Korea, Roman Catholicism spread across the Korean Peninsula. Protestant Christianity, which was introduced in the late nineteenth century, not only shared the Gospel but also contributed to the westernization of Korea, while introducing Anglo-Protestant civilization (12). With the explosive increase in Protestant Christian population and churches in Korea, Protestantism has also influenced Korean political and social life (13), making Korea the only East Asian nation where Christianity spread rapidly (14). On the other hand, more than half of the Koreans are irreligious.

Religion is expected to have a direct or indirect effect on various aspects of human life; therefore, it is also assumed to be associated with clinical characteristics and outcomes in patients with AMI. Despite various clinical studies on the risk factors and clinical outcomes of patients with AMI, evidence on the association between religion and AMI is limited. Despite several studies about the relationship between AMI and religion (8, 15–20), the beneficial effects of religions remain controversial due to the uncritical endorsement and lack of scientific rigor. To fill these gaps, we aimed to investigate the impact of religion on clinical outcomes in patients with AMI.

## Materials and Methods

### Study Design and Population

**FIGURE 1:**
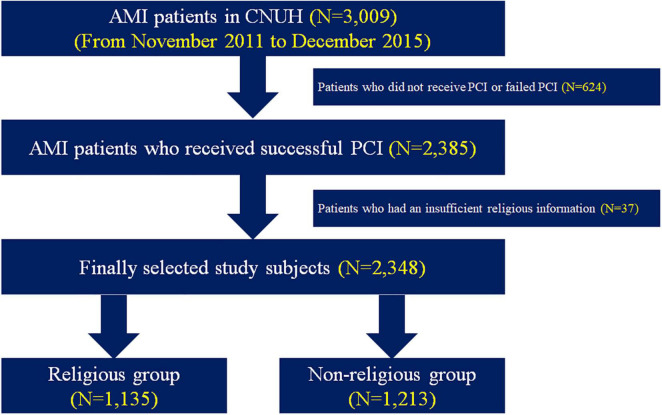
Flowchart of study population. AMI, acute myocardial infarction; CNUH, Chonnam National University Hospital; and PCI, percutaneous coronary intervention.

The study scheme is briefly illustrated in Figure 1. From November 2011 to December 2015, a total of 3,009 patients with AMI from Chonnam National University Hospital (CNUH) were initially selected. From this population, we chose patients who underwent successful percutaneous coronary intervention (PCI; *n* = 2,385). After excluding patients whose religious belief was unknown (*n* = 37), a total of 2,348 patients with AMI treated by PCI were finally enrolled in the present study. All these patients were subdivided into two groups depending on their religious faith (i.e., religious group and non-religious group).

### Definition

According to some contemporary guidelines (21, 22), AMI is a medical condition characterized by necrosis of cardiac myocytes and a rise and/or fall in the level of myocardial biomarkers, with the following clinical evidence of myocardial ischemia: (1) clinical symptoms and/or signs of myocardial ischemia, (2) newly detected ischemic change in the electrocardiogram (ECG) including ST-segment deviation and/or the development of pathologic Q-waves, (3) clinical evidence of new disappearance of viable myocardial segments or new abnormalities in regional wall motion from cardiovascular imaging modalities, and (4) identification of any coronary thrombus by coronary angiogram (CAG). According to some contemporary guidelines (21, 22), ST-segment elevation myocardial infarction (STEMI) was defined as a new-onset ST-segment elevation in ≥ 2 continuous leads, > 0.2 mV in precordial leads V1-3, or > 0.1 mV in all other leads on 12-lead ECG, with clinical evidence of AMI.

All patients with AMI were subdivided based on the Killip classification at presentation (23). Off-hour presentation was defined as arrival at the hospital during the weekend (Saturday and Sunday), national holidays, and night shifts (18:01–07:59) on weekdays. Body mass index (BMI) was measured using each patient’s weight and height. Angiographic and procedural characteristics were recorded and reviewed. Image-guided PCI refers to the utilization of optical coherence tomography or intravascular ultrasonography during the PCI procedure, and an infarct-related artery (IRA) is defined as an epicardial coronary artery that is occluded or stenosed by an atheromatous or thrombotic process, which is directly responsible for acute coronary syndrome. Coronary artery flow was classified based on the Thrombolysis In Myocardial Infarction (TIMI) flow grade (24), and LVEF was recorded *via* two-dimensional echocardiography.

### Data Collection

The medical history of all participants was routinely analyzed during hospitalization: an experienced nurse and attending physician were involved in this process to ensure that useful clinical information on demographics and previous medical, social, and family history was collected. Previous medical history includes hypertension, diabetes mellitus, dyslipidemia, myocardial infarction, heart failure, and cerebrovascular accident (CVA). During this process, each patient’s religious affiliation was also routinely checked and updated in the medical records. If it is difficult to obtain sufficient clinical information from the participants, we also appropriately referenced or utilized the responses of their caregivers. These data were collected into the structured and systematic survey format embedded in the electrical medical records.

The medical records of all patients were extensively and carefully reviewed during the study period. Data on baseline characteristics, prescribed medications, and angiographic and echocardiographic profiles were secured by full-time internal medicine residents and cardiovascular specialists.

### Clinical Outcomes

Follow-up after discharge from the index hospitalization was performed for 12 months. The primary outcome was the occurrence of major adverse cardiac and cerebrovascular events (MACCEs). MACCE refers to the composition of all-cause death (including cardiac and non-cardiac death), non-fatal myocardial infarction (NFMI), any revascularization (i.e., any re-do PCI or coronary artery bypass grafting), rehospitalization due to angina, CVA, and stent thrombosis. The secondary outcomes included net adverse clinical event (NACE), all-cause death, cardiac and non-cardiac death, NFMI, any revascularization, rehospitalization due to angina, CVA, and stent thrombosis. NACE refers to the composition of cardiac death, NFMI, any revascularization, and rehospitalization due to angina.

### Statistical Analysis

Categorical variables, expressed as the number of cases with percentages, were compared using the chi-squared test or Fisher’s exact test. Continuous variables, expressed as mean ± standard deviation, were compared using Student’s *t*-test, one-way analysis of variance, or Kruskal–Wallis test. The survival analysis of all clinical outcomes such as MACCE, NACE, and all-cause death according to the two groups (religious and non-religious groups) was conducted using a log-rank test. All the results of this study were rendered significant at a *p*-value < 0.05.

To minimize the effect of selection bias on the analysis of the observational cohort, we used two propensity score-weighting methods: the propensity score matching (PSM) and the inverse probability of treatment weighting (IPTW). In these methods, we formulated the propensity score using a multivariable logistic regression analysis with a total of 30 covariates. These covariates included sex, age, utilization of emergency medical service, symptom-to-door time (S2DT), door-to-balloon time (D2BT), total ischemic time (TIT), hospital visit timings (off-hour vs. on-hour admission), Killip classification, BMI, six components of previous medical history, smoking history, family history of coronary artery disease, prescribed medications (such as aspirin, P2Y12 inhibitors, beta-blockers, angiotensin-converting enzyme inhibitors/angiotensin receptor blockers, and statins), vascular approach, use of glycoprotein IIb/IIIa inhibitors, use of thrombus aspiration, use of image-guided PCI, IRA (left main coronary artery, left anterior descending coronary artery vs. left circumflex coronary artery, and right coronary artery), preprocedural TIMI flow grade, use of thrombolysis, and LVEF. Furthermore, patients who lacked data on these covariates or those with a follow-up interval after hospital discharge of 0 days were excluded from the PSM- and IPTW-adjusted analyses.

We analyzed and compared 12-month clinical outcomes and landmark analysis from 90 days between the religious and non-religious groups using a Kaplan–Meier curve with a log-rank test. We also calculated the hazard ratios and 95% confidence intervals for 12-month clinical outcomes using Cox proportional hazards regression models.

All analyses were performed using SPSS version 25.0 (IBM Corp., Armonk, NY, United States).

### Ethical Approval

This study complied with the ethical standards of the Helsinki Declaration, which was finally revised in 2013 (25). The study protocol was extensively reviewed and approved by the Institutional Review Board (IRB) of CNUH (IRB No. CNUH-2021-403), and the requirement for informed consent was waived because of this study’s retrospective nature.

## Results

### Baseline Clinical and Procedural Characteristics

**FIGURE 2:**
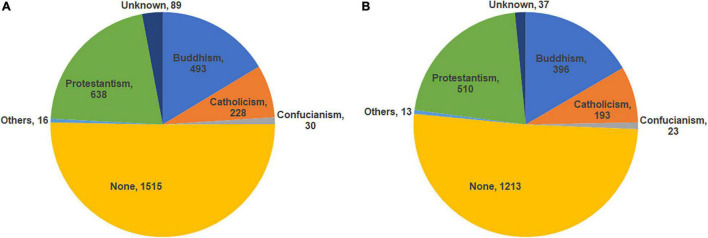
Religious distribution of the study population. **(A)** AMI patients who underwent successful PCI (*n* = 2,385). **(B)** Finally selected study patients (*n* = 2,348).

The religious distribution of all study participants is detailed in Figure 2. In the overall study population, there was a predominance of patients without religious affiliation. In the religious group, Protestantism was the predominant religion, followed by Buddhism and Catholicism.

Baseline characteristics of the study population are summarized in Table 1. Overall, 1,135 (48.3%) of the 2,348 patients with AMI belonged to the religious group, while the rest of them (51.7%) belonged to the non-religious group. Compared to the religious group, patients in the non-religious group were younger, mostly men, more likely to smoke, and more likely to be diagnosed with STEMI. Additionally, the non-religious group was less likely to have a history of hypertension and tended to receive PCI more quickly with shorter D2BT. As shown in Table 2, the coronary angiographic and procedural characteristics were not significantly different between the two groups. After adjustment with PSM and IPTW, all differences were well-balanced (Tables 1, 2).

**TABLE 1:** Baseline characteristics of the patients.

	Before propensity score weighting method			After PSM			After IPTW		
			
Characteristics	Religious group	Non-religious group	*p*-value	Religious group	Non-religious group	*p*-value	Religious group	Non-religious group	*p*-value
					
	(*n* = 1,135)	(*n* = 1,213)		(*n* = 910)	(*n* = 910)		(*n* = 2,313)	(*n* = 2,312)	
Male patients	712 (62.7)	973 (80.2)	<0.001	672 (73.8)	674 (74.1)	0.915	1,662 (71.8)	1,662 (71.9)	0.986
Age ≥ 65 years	668 (58.9)	604 (49.8)	<0.001	480 (52.7)	503 (55.3)	0.279	1,248 (54.0)	1,247 (53.9)	0.985
EMS utilization	107 (8.8)	95 (8.4)	0.811	77 (8.5)	82 (9.0)	0.678	201 (8.7)	199 (8.6)	0.939
TIT ≥ 12 h	591 (52.1)	595 (49.1)	0.144	447 (49.1)	471 (51.8)	0.261	1,165 (50.4)	1,166 (50.4)	0.970
S2DT ≥ 4 h	184 (16.2)	182 (15.0)	0.420	138 (15.2)	150 (16.5)	0.441	358 (15.5)	359 (15.5)	0.971
D2BT ≥ 90 min	692 (61.0)	687 (56.6)	0.033	532 (58.5)	552 (60.7)	0.339	1,353 (58.5)	1,351 (58.4)	0.970
Killip class III-IV	136 (12.0)	134 (11.0)	0.478	97 (10.7)	107 (11.8)	0.457	263 (11.4)	262 (11.3)	0.983
BMI ≥ 25 kg/m^2^	409 (36.3)	421 (34.9)	0.485	327 (35.9)	334 (36.7)	0.733	824 (35.6)	825 (35.7)	0.980
Previous history									
Hypertension	667 (58.8)	595 (49.1)	<0.001	492 (54.1)	516 (56.7)	0.258	1,244 (53.8)	1,245 (53.8)	0.991
Diabetes mellitus	363 (32.0)	366 (30.2)	0.344	273 (30.0)	294 (32.3)	0.288	718 (31.0)	717 (31.0)	0.979
Dyslipidemia	93 (8.2)	83 (6.8)	0.214	68 (7.5)	74 (8.1)	0.600	175 (7.6)	177 (7.7)	0.938
Old MI	89 (7.8)	105 (8.7)	0.474	76 (8.3)	68 (7.5)	0.487	190 (8.2)	190 (8.2)	0.984
Old heart failure	15 (1.3)	18 (1.5)	0.738	13 (1.4)	13 (1.4)	1.000	30 (1.3)	31 (1.3)	0.953
Old CVA	74 (6.5)	79 (6.5)	0.994	63 (6.9)	58 (6.4)	0.638	152 (6.6)	153 (6.6)	0.976
Smoking	569 (50.1)	813 (67.0)	<0.001	539 (59.2)	529 (58.1)	0.634	1,361 (58.9)	1,362 (58.9)	0.988
Family history of CAD	62 (5.5)	61 (5.0)	0.637	47 (5.2)	54 (5.9)	0.474	122 (5.3)	120 (5.2)	0.928
LVEF < 40%	120 (10.6)	124 (10.3)	0.800	80 (9.6)	95 (10.4)	0.532	240 (10.4)	240 (10.4)	0.997
STEMI diagnosis	451 (39.7)	538 (44.4)	0.024	385 (42.3)	367 (40.3)	0.392	977 (42.2)	980 (42.4)	0.944
Discharge medications									
Aspirin	1,134 (99.9)	1,212 (99.9)	0.962	909 (99.9)	909 (99.9)	1.000	2,311 (99.9)	2,311 (99.9)	1.000
P2Y12 inhibitors	1,134 (99.9)	1,212 (99.9)	0.962	909 (99.9)	910 (100.0)	1.000	2,311 (99.9)	2,311 (99.9)	1.000
Beta-blockers	981 (86.4)	1,041 (85.8)	0.669	789 (86.7)	785 (86.3)	0.784	1,995 (86.3)	1,993 (86.2)	0.971
ACE inhibitor or ARB	992 (87.4)	1,057 (87.1)	0.849	806 (88.6)	795 (87.4)	0.428	2,023 (87.5)	2,022 (87.4)	0.983
Statins	1,062 (93.6)	1,144 (94.3)	0.450	855 (94.0)	849 (93.3)	0.565	2,175 (94.0)	2,176 (94.1)	0.942

*Values are presented as number (percentage) for categorical values and means ± standard deviation for continuous variables. ACE, angiotensin-converting enzyme; ARB, angiotensin receptor blocker; BMI, body mass index; CAD, coronary artery disease; CrCl, creatinine clearance; CVA, cerebrovascular accidents; EMS, emergency medical service; IPTW, inverse probability of treatment weighting; LVEF, left ventricular ejection fraction; MI, myocardial infarction; PCI, percutaneous coronary intervention; PSM, propensity score matching.*

**TABLE 2:** Coronary angiographic and procedural characteristics of the study population.

	Before propensity score weighting			After PSM			After IPTW		
			
Characteristics	Religious group	Non-religious group	*p*-value	Religious group	Non-religious group	*p*-value	Religious group	Non-religious group	*p*-value
					
	(*n* = 1,135)	(*n* = 1,213)		(*n* = 910)	(*n* = 910)		(*n* = 2,313)	(*n* = 2,312)	
Use of transfemoral approach	602 (53.0)	669 (55.2)	0.305	492 (54.1)	471 (51.8)	0.324	1,243 (53.7)	1,244 (53.8)	0.968
Use of GPIIb/IIIa inhibitor	159 (14.0)	169 (13.9)	0.957	123 (13.5)	141 (15.5)	0.231	326 (14.1)	323 (14.0)	0.938
Use of thrombus aspiration	176 (15.5)	219 (18.1)	0.099	147 (16.1)	161 (17.7)	0.381	395 (17.1)	391 (16.9)	0.925
Use of image-guided PCI	51 (4.5)	76 (6.3)	0.058	47 (5.2)	40 (4.4)	0.442	129 (5.6)	126 (5.4)	0.892
LMCA or LAD as an IRA	556 (49.0)	590 (48.6)	0.866	446 (49.0)	440 (48.3)	0.778	1,139 (49.2)	1,141 (49.3)	0.956
Use of thrombolysis	1 (0.1)	3 (0.2)	0.626	1 (0.1)	1 (0.1)	1.000	3 (0.1)	4 (0.2)	0.742
Preprocedural TIMI flow grade 0-I	558 (49.2)	636 (52.4)	0.113	471 (51.8)	452 (49.7)	0.373	1,185 (51.2)	1,189 (51.4)	0.941

*Values are presented as number (percentage) for categorical values and means ± standard deviation for continuous variables. GPIIb/IIIa, glycoprotein IIb/IIIa; IPTW, inverse probability of treatment weighting; LAD, left anterior descending coronary artery; LMCA, left main coronary artery; PCI, percutaneous coronary intervention; PSM, propensity score matching; TIMI, Thrombolysis in Myocardial Infarction.*

### Clinical Outcomes

**FIGURE 3:**
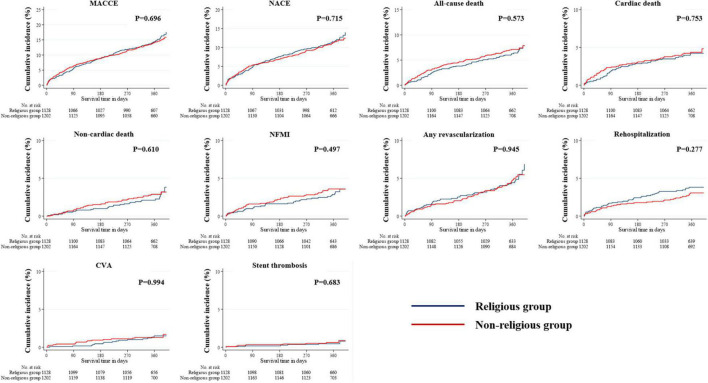
Incidences of primary and secondary clinical outcomes in all patients after a 1-year follow-up (before PSM- or IPTW-adjusted analysis). The figure shows the Kaplan–Meier curves for cumulative event rates according to the presence or absence of religious faith. IPTW, inverse probability of treatment weighting; PSM, propensity score matching.

**FIGURE 4:**
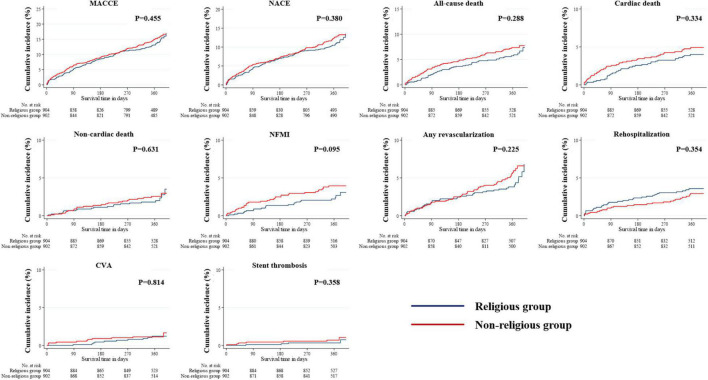
Incidences of primary and secondary clinical outcomes in all patients after a 1-year follow-up (after PSM-adjusted analysis). The figure shows the Kaplan–Meier curves for the cumulative event rates according to the presence or absence of religious faith. PSM, propensity score matching.

**FIGURE 5:**
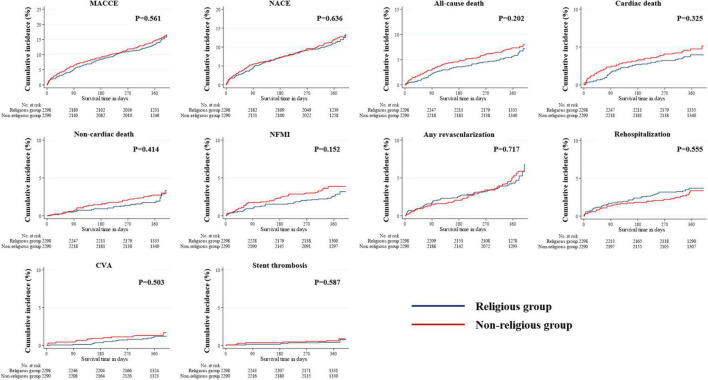
Incidences of primary and secondary clinical outcomes in all patients after a 1-year follow-up (after IPTW-adjusted analysis). The figure shows the Kaplan–Meier curves for the cumulative event rates according to their religious faith. IPTW, inverse probability of treatment weighting.

The median follow-up period in the study population was 363 days. All outcomes, which were evaluated for 12 months, were determined, including MACCE, NACE, all-cause death, cardiac and non-cardiac death, NFMI, any revascularization, CVA, and stent thrombosis (Table 3). The Kaplan–Meier survival analysis was used to plot the unadjusted, PSM-adjusted, and IPTW-adjusted survival curves (Figures 3–5). Before and after PSM and IPTW adjustment, no significant differences in any clinical outcomes between the religious and non-religious groups were found.

**TABLE 3:** One-year clinical outcomes in propensity score matched post-discharge survivors.

Outcomes	Religious group	Non-religious group	Unadjusted analysis		PSM-adjusted analysis		IPTW-adjusted analysis	
					
	(*n* = 1,128)	(*n* = 1,202)	HR (95% CI) (a)	*p*-value	HR (95% CI) (b)	*p*-value	HR (95% CI) (b)	*p*-value
MACCE (c)	176 (15.6)	184 (15.3)	0.975 (0.793–1.199)	0.811	1.113 (0.878–1.410)	0.377	1.077 (0.869–1.336)	0.497
NACE	138 (12.2)	144 (12.0)	0.968 (0.766–1.222)	0.783	1.134 (0.870–1.477)	0.352	1.067 (0.838–1.358)	0.598
All-cause death	76 (6.7)	89 (7.4)	1.093 (0.805–1.485)	0.568	1.217 (0.854–1.733)	0.278	1.237 (0.894–1.711)	0.199
Cardiac death	48 (4.3)	54 (4.5)	1.049 (0.711–1.548)	0.809	1.249 (0.805–1.937)	0.321	1.214 (0.807–1.826)	0.353
Non-cardiac death	28 (2.5)	35 (2.9)	1.169 (0.711–1.922)	0.538	1.158 (0.636–2.109)	0.631	1.280 (0.750–2.187)	0.366
NFMI	36 (3.2)	42 (3.5)	1.086 (0.695–1.695)	0.718	1.418 (0.840–2.393)	0.191	1.291 (0.815–2.045)	0.276
Any revascularization	56 (5.0)	63 (5.2)	1.028 (0.717–1.474)	0.880	1.320 (0.884–1.972)	0.175	1.110 (0.768–1.604)	0.578
Rehospitalization due to angina	42 (3.7)	34 (2.8)	0.757 (0.482–1.190)	0.228	0.750 (0.442–1.274)	0.288	0.843 (0.529–1.345)	0.474
CVA	17 (1.5)	17 (1.4)	0.926 (0.473–1.814)	0.822	1.109 (0.471–2.610)	0.814	1.153 (0.572–2.322)	0.690
Stent thrombosis	6 (0.5)	10 (0.8)	1.429 (0.516–3.957)	0.492	1.971 (0.598–6.495)	0.265	1.517 (0.548–4.204)	0.423

*Values are presented as percentage (number) for categorical values.*

*BMI, body mass index; CI, confidence interval; CVA, cerebrovascular accident; D2BT, door-to-balloon time; HR, hazard ratio; IPTW, inverse probability of treatment weighting; IRA, infarct-related artery; LAD, left anterior descending coronary artery; LCX, left circumflex coronary artery; LMCA, left main coronary artery; LVEF, left ventricular ejection fraction; MACCE, major adverse cardiac and cerebrovascular events; NACE, net adverse clinical events; NFMI, non-fatal myocardial infarction; PCI, percutaneous coronary intervention; PSM, propensity score matching; RCA, right coronary artery; S2DT, symptom-to-door time; TIMI, Thrombolysis In Myocardial Infarction; TIT, total ischemic time.*

*(a) HR corresponds to the non-religious group compared with the religious group. (b) The adjusted Cox hazards regression analysis included various clinical variables, including sex, age, utilization of emergency medical service, S2DT, D2BT, TIT, hospital visit timing (off-hour vs. on-hour admission), Killip classification, BMI, previous medical history (hypertension, diabetes mellitus, dyslipidemia, old myocardial infarction, prior heart failure, old CVA), smoking history, family coronary artery disease history, prescribed medications (aspirin, P2Y12 inhibitors, beta-blockers, angiotensin-converting enzyme inhibitors/angiotensin receptor blockers, and statins), vascular approach, use of glycoprotein IIb/IIIa inhibitors, use of thrombus aspiration, use of image-guided PCI, IRA (LMCA or LAD vs. LCX or RCA), preprocedural TIMI flow grade, use of thrombolysis, and LVEF. (c) MACCE is defined as a composite of all-cause mortality, non-fatal myocardial infarction, any revascularization, cerebrovascular accident, and stent thrombosis.*

**FIGURE 6:**
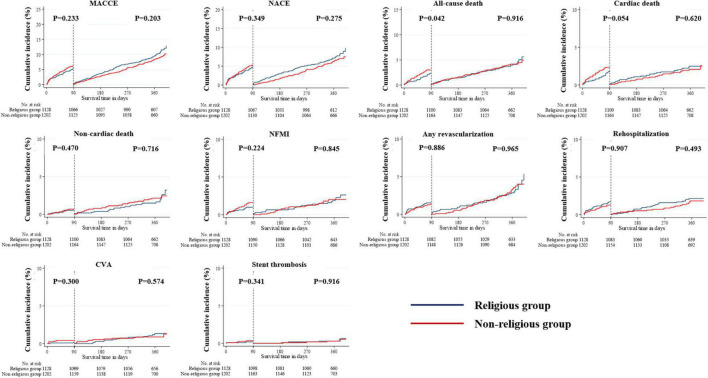
Incidences of primary and secondary clinical outcomes for all patients in a landmark analysis between 90 days and 1 year (before PSM- or IPTW-adjusted analysis). The figure shows the Kaplan–Meier curves for the cumulative event rates according to their religious faith. IPTW, inverse probability of treatment weighting; PSM, propensity score matching.

**FIGURE 7:**
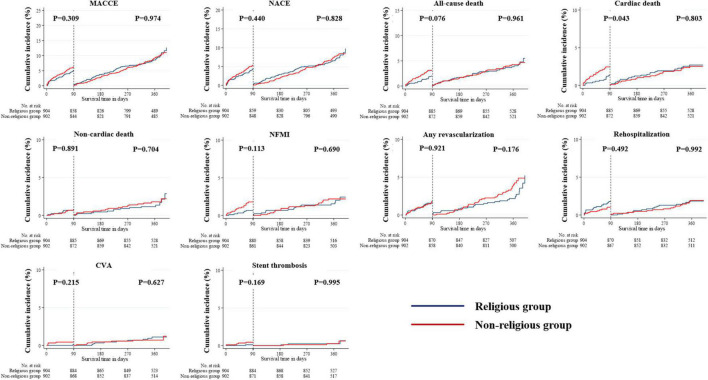
Incidences of primary and secondary clinical outcomes for all patients in a landmark analysis between 90 days and 1 year (after PSM-adjusted analysis). The figure shows the Kaplan–Meier curves for the cumulative event rates according to their religious faith. PSM, propensity score matching.

**FIGURE 8:**
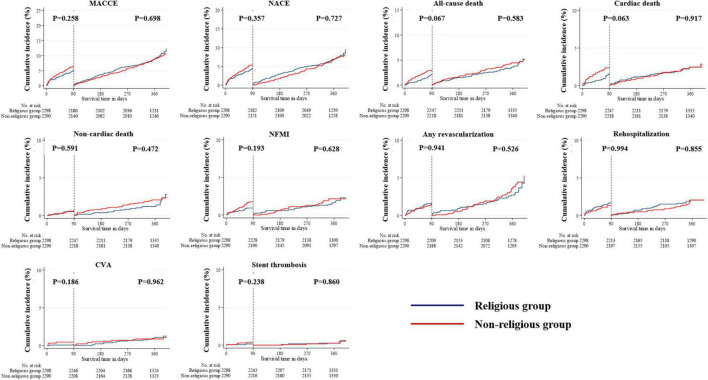
Incidences of primary and secondary clinical outcomes for all patients in a landmark analysis between 90 days and 1 year (after IPTW-adjusted analysis). The figure shows the Kaplan–Meier curves for the cumulative event rates according to their religious faith. IPTW, inverse probability of treatment weighting.

The landmark analysis was also conducted in all unadjusted, PSM- and IPTW-adjusted cohorts (Figures 6–8). All-cause death within the first 90 days in the unadjusted cohort (*p* = 0.042) was significantly higher in the non-religious group than in the religious group and tended to be higher in the non-religious group than in the religious group among the two adjusted cohorts, respectively (*p* = 0.076 for PSM-adjusted samples, *p* = 0.067 for IPTW-adjusted samples). Cardiac death within the first 90 days in the PSM-adjusted cohorts (*p* = 0.043) was significantly higher in the non-religious group than in the religious group, while that in IPTW-adjusted cohorts tended to be higher in the non-religious group than in the religious group (*p* = 0.063).

## Discussion

We performed a comparative analysis of clinical outcomes among patients with AMI with or without religious affiliation. All clinical data of the 2,348 patients in the study population were analyzed. As for religious distribution, patients without religious affiliation constituted the largest portion of the study population. In the religious group, Protestantism ranked first among other faiths, followed by Buddhism and Catholicism. Considering that Christianity and Buddhism are the dominant confessions of faith with the prosperity of Protestant Christianity in Korea (11, 12), it appears that the religious distribution of the study population is similar to that of the entire population of Korea.

The patients of the non-religious group tended to be younger and were mostly men, which also had higher proportions of smokers and patients with a diagnosis of STEMI. Traditionally, male sex and smoking have been recognized as risk factors of myocardial infarction (26, 27). Generally, STEMI is considered a more emergent medical condition than non-STEMI (NSTEMI) and requires a faster reperfusion strategy called primary PCI. For this reason, D2BT appears to be shorter in the non-religious group than in the religious group.

In addition, the non-religious group had a lower proportion of a history of hypertension than the religious group. A detailed investigation of the age distribution of the overall study population is summarized in Supplementary Table 1. Patients with AMI aged < 60 years were more prevalent in the non-religious group, while sexagenarian, septuagenarian, octogenarian, and nonagenarian patients were more predominant in the religious group. Therefore, the lower prevalence of hypertension in the non-religious group is best explained by the fact that hypertension is less prevalent in young ages, according to a study by Kang et al. (28).

Meanwhile, another characteristic feature we were interested in was the significant difference in smoking patterns between the two groups, in line with several epidemiologic studies showing negative relationship between religiosity and the likelihood of smoking (29, 30). Although behavioral restrictions vary considerably among religions, major world religions either do not approve the abuse of tobacco smoking or at least have positions that are opposed to smoking behavior (31, 32). Christianity tends to welcome anti-smoking behaviors according to the Meeting on Tobacco and Religion held in Geneva, Switzerland, in May 1999 (31). In Buddhism, since tobacco tends to be considered a harmful and addictive substance, religious friars are advised to refrain from tobacco use, but smoking is not entirely prohibited (31). These religious ethics and limitations on tobacco use may have contributed directly or indirectly to the relatively lower smoking history of the patients in the religious group than those in the non-religious group.

Despite some notable differences in baseline characteristics, the 1-year clinical outcomes in both groups were comparable. These results can be explained as follows: First, the similarity in the procedural characteristics in both groups ensures that the two groups received PCI procedures of similar quality. Second, as part of the post-PCI management, both groups received the same discharge medications. Third, despite the difference in D2BT, TIT was comparable in both groups, which was mainly attributed to the similar S2DT. The similar trend in S2DT appeared to be partially contributed by the comparable EMS utilization rate. Taken together, no significant differences were noted in the clinical outcomes, irrespective of religion, thanks to the multifaceted and comprehensive management for AMI.

Although no difference was found between the two groups, in our additional landmark analysis, the religious group appeared to have more favorable outcomes in terms of all-cause death and cardiac death within the first 90 days than the non-religious group. Notably, a significant difference was observed in cardiac death within the first 90 days in the PSM-adjusted cohorts. These findings seem to be interesting; however, the reason for this finding remains unclear. If we consider that religion is related to some major risk factors for AMI such as lifestyle modification and medication adherence (33), religion appears to have provoked enhanced and intensive awareness of post-AMI management, influencing good outcomes in the short-term interval; however, further investigation is necessary. Nevertheless, through the application of multifaceted treatments, including the maintenance of pharmacological therapy, this positive effect of religiosity appears to diminish over time, which is reflected in the statistical attenuation with respect to clinical outcomes after 90 days.

Although PSM- and IPTW-adjusted analyses showed non-significant results for each outcome variable, the non-religious group demonstrated slight but non-significant trends of higher incidences of most of the outcome variables, except for rehospitalization, than the religious group. Rehospitalization consistently tended to show a slightly higher incidence in the religious group than in the non-religious group in both unadjusted and adjusted analyses. This contrasting trend between rehospitalization and the rest of the outcome variables may be one of the interesting aspects. According to a clinical study on post-AMI rehospitalization, age is negatively associated with rehospitalization for acute coronary syndrome and revascularization and that for unstable angina (34). Therefore, the difference in age between the patients of religious and non-religious groups may have influenced this tendency.

Apart from the main subject of this study, we also investigated in-hospital outcomes in both groups. Data are presented in Supplementary Table 2. Most in-hospital complications were comparable in both groups, except for cardiogenic shock. The incidence of cardiogenic shock was slightly higher in the non-religious group than in the religious group, which may be because the non-religious group had a larger percentage of STEMI patients and STEMI generally has worse short-term outcomes than NSTEMI (35). Nonetheless, in-hospital death was comparable between the two groups (0.6% in the religious group and 0.9% in the non-religious group).

Religion plays a crucial role in everyday human life. There is a gradually increasing interest in the health benefits of religious beliefs, and there has been a common belief that religious involvement can promote both physical and mental well-being. Through comprehensive and extensive literature review, published studies on the various effects of religion on human heath were identified (7), which include decreased morbidity and mortality risk, better recovery, and better quality of life (9). According to Koenig, more than 850 articles on religious behavior and mental health have been published and 350 articles on religious behavior and physical health have been published (36). Almost all published studies have emphasized that religious activities have beneficial effects on human health (7, 9, 37).

Nevertheless, there has been a paucity of information on the association between religious activities and myocardial infarction, except for a few published articles (8, 15–17). Momennasab et al. noted the positive effects of spirituality in nine hospitalized Muslim patients with AMI (15). Roshi et al. observed no difference in the incidence of myocardial infarction between two different religious groups (Christianity and Islam) (16). Kamm-Steigelman et al. found that religion serves as a coping resource, providing strength and comfort during the recovery of female patients with AMI (17). However, these three studies are indeed not prospective cohort studies and also have small sample sizes. On the other hand, a study conducted by Blumenthal and his colleagues is a prospective cohort study with a relatively large number of participants, which showed little evidence of the relationship between religious activities and the morbidity and mortality post AMI (8). To the best of our best knowledge, this is the first comparative study on clinical characteristics and outcomes in Korean patients with AMI in relation to their religious belief. Our results demonstrated that religion did not affect the clinical prognosis of patients with AMI, despite some notable clinical features in the baseline characteristics and favorable trend for a short time period.

However, this study has several limitations. First, this study was conducted in a single tertiary medical institution. Thus, it is difficult to generalize the clinical characteristics and outcomes including the incidence of MACCE, all-cause death, and cardiac death from this study to the overall AMI population. Second, this study is a non-randomized and retrospective with an observational cohort. In reality, it is difficult to a conduct randomized clinical study on religious affiliation. Thus, although two propensity score-weighing methods, such as PSM and IPTW, were performed to reduce selection bias, a multicenter study is needed in the future. Third, although our results demonstrated similar trends with the religious distribution of the overall Korean population (Figure 2), this study did not sufficiently reflect other major religions of the world. In other words, our study did not include major world religions such as Eastern Orthodoxy, Hinduism, Islam, or Judaism. Fourth, despite the notable value of the results of this study, religious assessments were only conducted through a questionnaire survey and a multifaceted evaluation of religious fidelity or depth of religious faith such as self-reported spirituality, frequency of visits or attendance to religious shrines, or frequency of religious practice was not performed with lack of quantitative analysis. In other words, various elements of religiosity were lacking, but only religious affiliation was included in the present study, which suggests that there are still a lot of things to explain about the influence of religion on the results of our study.

In summary, despite a growing body of evidence that religious activities have better effects on human physical health, this study did not reveal significant differences in the 1-year clinical outcomes in patients with AMI irrespective of religious beliefs.

## Data Availability Statement

The raw data supporting the conclusions of this article will be made available by the authors, without undue reservation.

## Ethics Statement

The studies involving human participants were reviewed and approved by the Institutional Board Review of Chonnam National University Hospital. Written informed consent for participation was not required for this study in accordance with the national legislation and the institutional requirements.

## Author Contributions

SO conceptualized the work, performed formal analysis, carried out software processes, and wrote the original draft of this manuscript. SO, KC, MK, DS, and YH curated the data. SO and JK performed investigation. KC, MK, DS, YH, JK, YA, and MJ edited and reviewed the manuscript. All authors contributed to the article and approved the submitted version.

## Conflict of Interest

The authors declare that the research was conducted in the absence of any commercial or financial relationships that could be construed as a potential conflict of interest.

## Publisher’s Note

All claims expressed in this article are solely those of the authors and do not necessarily represent those of their affiliated organizations, or those of the publisher, the editors and the reviewers. Any product that may be evaluated in this article, or claim that may be made by its manufacturer, is not guaranteed or endorsed by the publisher.

## Abbreviations

AMI: acute myocardial infarction
BMI: body mass index
CAG: coronary angiography
CNUH: Chonnam National University Hospital
CVA: cerebrovascular accident
D2BT: door-to-balloon time
ECG: electrocardiogram
IPTW: inverse probability of treatment weighting
IRA: infarct-related artery
LAD: left anterior descending coronary artery
LCX: left circumflex coronary artery
LMCA: left main coronary artery
LVEF: left ventricular ejection fraction
MACCE: major adverse cardiac and cerebrovascular event
NACE: net adverse clinical event
NFMI: non-fatal myocardial infarction
PCI: percutaneous coronary intervention
PSM: propensity score matching
RCA: right coronary artery
S2DT: symptom-to-door time
STEMI: ST-segment elevation myocardial infarction
TIMI: Thrombolysis In Myocardial Infarction
TIT: total ischemic time.
