# Supplementary material for: Religious Affiliations and Clinical Outcomes in Korean Patients With Acute Myocardial Infarction
Source: Front Cardiovasc Med. 2022 Mar 23;9:835969. doi: 10.3389/fcvm.2022.835969 (PMC8984284; doi:10.3389/fcvm.2022.835969)
Supplement: Supplementary file 1 [file Table_1.DOCX]

**Supplemental Table 1**. Age distribution of the overall study population

| Characteristics | Religious group | Non-religious group | *p*-value |
| --- | --- | --- | --- |
|  | **(n=1,135)** | **(n=1,213)** |  |
| Age group |  |  | **<0.001** |
| Age ≥ 65 years | 668 (58.9) | 604 (49.8) |  |
| Age ≥ 45 and < 65 years | 412 (36.3) | 525 (43.3) |  |
| Age < 45 years | 55 (4.8) | 84 (6.9) |  |
| Age subgroup |  |  |  |
| Age ≥ 20 and < 30 years | 0 (0.0) | 3 (0.2) |  |
| Age ≥ 30 and < 40 years | 17 (1.5) | 22 (1.8) |  |
| Age ≥ 40 and < 50 years | 101 (3.8) | 171 (14.1) |  |
| Age ≥ 50 and < 60 years | 215 (18.9) | 283 (23.3) |  |
| Age ≥ 60 and < 70 years | 272 (24.0) | 285 (23.5) |  |
| Age ≥ 70 and < 80 years | 358 (31.5) | 322 (26.5) |  |
| Age ≥ 80 and < 90 years | 159 (14.0) | 119 (9.8) |  |
| Age ≥ 90 years | 13 (1.1) | 8 (0.7) |  |

Values are presented as number with percentage for categorical values.
