# Supplementary material for: Religious Affiliations and Clinical Outcomes in Korean Patients With Acute Myocardial Infarction
Source: Front Cardiovasc Med. 2022 Mar 23;9:835969. doi: 10.3389/fcvm.2022.835969 (PMC8984284; doi:10.3389/fcvm.2022.835969)
Supplement: Supplementary file 2 [file Table_2.DOCX]

**Supplementary Table 2**. In-hospital outcomes during index hospitalization, from 2,348 AMI with or without any religious affiliation.

| Characteristics | Religious group  (n = 1,135) | Non-religious group  (n = 1,213) | *p*-value |
| --- | --- | --- | --- |
| In-hospital death | 7 (0.6) | 11 (0.9) | 0.421 |
| In-hospital complications |  |  |  |
| Cardiogenic shock | 76 (6.7) | 106 (8.7) | 0.064 |
| New-onset heart failure | 83 (7.3) | 80 (6.6) | 0.494 |
| Non-fatal myocardial infarction | 7 (0.6) | 7 (0.6) | 0.901 |
| Stent thrombosis | 7 (0.6) | 7 (0.6) | 0.901 |
| Ischemic or hemorrhagic CVA | 10 (0.9) | 16 (1.3) | 0.311 |
| Bleeding complications  (Hgb decrease by 5 g/dL or Hct decrease by 15 %) | 16 (1.4) | 18 (1.5) | 0.880 |
| Atrioventricular block | 35 (3.1) | 40 (3.3) | 0.768 |
| Ventricular tachycardia or fibrillation | 51 (4.5) | 72 (5.9) | 0.117 |
| Atrial fibrillation | 45 (4.0) | 39 (3.2) | 0.328 |
| Acute kidney injury | 5 (0.4) | 7 (0.6) | 0.643 |
| Sepsis | 6 (0.5) | 7 (0.6) | 0.874 |
| Supportive treatment |  |  |  |
| CPR | 62 (5.5) | 63 (5.2) | 0.772 |
| Percutaneous ventricular mechanical support devices |  |  |  |
| IABP | 27 (2.4) | 29 (2.4) | 0.985 |
| ECMO | 2 (0.2) | 2 (0.2) | 1.000 |
| Any defibrillation | 39 (3.4) | 52 (4.3) | 0.286 |
| Permanent pacemaker implantation | 2 (0.2) | 2 (0.2) | 1.000 |
| ICD | 2 (0.2) | 3 (0.2) | 1.000 |
| CABG | 1 (0.1) | 1 (0.1) | 1.000 |

Values are presented as number (percentage) for categorical values.

CABG = coronary artery bypass graft; CPR = cardiopulmonary resuscitation; CVA = cerebrovascular accident; ECMO = extracorporeal membrane oxygenation; Hct = hematocrit; Hgb = hemoglobin; IABP = intra-aortic balloon pump; ICD = implantable cardiac defibrillator.
