# Supplementary material for: Religious Affiliations and Clinical Outcomes in Korean Patients With Acute Myocardial Infarction
Source: Front Cardiovasc Med. 2022 Mar 23;9:835969. doi: 10.3389/fcvm.2022.835969 (PMC8984284; doi:10.3389/fcvm.2022.835969)
Supplement: Supplementary file 3 [file Table_3.DOCX]

**Supplemental Table 3**. One-year clinical outcomes of the study population depending on the religious affiliation.

| Outcomes | Protestantism  (N = 510) | Buddhism  (N = 396) | Catholicism  (N = 193) | Confucianism  (N = 23) | Others  (N = 13) |
| --- | --- | --- | --- | --- | --- |
| MACCE^a)^ | 68 (13.3) | 63 (15.9) | 44 (22.8) | 2 (8.7) | 1 (7.7) |
| NACE | 51 (10.0) | 51 (12.9) | 36 (18.7) | 2 (8.7) | 0 (0.0) |
| All-cause death | 28 (5.5) | 24 (6.1) | 22 (11.4) | 2 (8.7) | 1 (7.7) |
| Cardiac death | 15 (2.9) | 15 (3.8) | 16 (8.3) | 2 (8.7) | 0 (0.0) |
| Non-cardiac death | 13 (2.5) | 9 (2.3) | 6 (3.1) | 0 (0.0) | 1 (7.7) |
| Non-fatal myocardial infarction | 15 (2.9) | 16 (4.0) | 6 (3.1) | 0 (0.0) | 0 (0.0) |
| Any revascularization | 24 (4.7) | 23 (5.8) | 9 (4.7) | 0 (0.0) | 0 (0.0) |
| Rehospitalization due to angina | 14 (2.7) | 16 (4.0) | 13 (6.7) | 0 (0.0) | 0 (0.0) |
| CVA | 5 (1.0) | 7 (1.8) | 5 (2.6) | 0 (0.0) | 0 (0.0) |
| Stent thrombosis | 4 (0.8) | 1 (0.3) | 1 (0.5) | 0 (0.0) | 0 (0.0) |

Values are presented as percentage (number) for categorical values.

CVA = cerebrovascular accident; MACCE = major adverse cardiac and cerebrovascular events; NACE = net adverse clinical events

**^a)^**MACCE is defined as a composite of all-cause mortality, non-fatal myocardial infarction, any revascularization, cerebrovascular accident, and stent thrombosis.
